# Automatic Three-Dimensional Measurement of Large-Scale Structure Based on Vision Metrology

**DOI:** 10.1155/2014/185269

**Published:** 2014-02-17

**Authors:** Zhaokun Zhu, Banglei Guan, Xiaohu Zhang, Daokui Li, Qifeng Yu

**Affiliations:** ^1^College of Aerospace Science and Engineering, National University of Defense Technology, Changsha 410073, China; ^2^Hunan Provincial Key Laboratory of Image Measurement and Vision Navigation, National University of Defense Technology, Changsha 410073, China

## Abstract

All relevant key techniques involved in photogrammetric vision metrology for fully automatic 3D measurement of large-scale structure are studied. A new kind of coded target consisting of circular retroreflective discs is designed, and corresponding detection and recognition algorithms based on blob detection and clustering are presented. Then a three-stage strategy starting with view clustering is proposed to achieve automatic network orientation. As for matching of noncoded targets, the concept of matching path is proposed, and matches for each noncoded target are found by determination of the optimal matching path, based on a novel voting strategy, among all possible ones. Experiments on a fixed keel of airship have been conducted to verify the effectiveness and measuring accuracy of the proposed methods.

## 1. Introduction

As science and technology develop, more and more large-scale structures come into our lives, such as bridges, tunnels, airplanes, airships, wind turbine blades, and antennas. To ensure safety and prevent potential accidents and disasters, it is necessary to conduct mechanical testing and dimensional quality monitoring for these large-scale structures, which require automatic three-dimensional measurement with high metric precision.

Deformation measurement of large-scale structures constitutes a 3D coordinate measuring problem involving large measurement ranges and high measuring accuracy. Compared to other measuring means such as strain gauge method [1, 2], surveying method [3, 4], and GPS method [5–7], photogrammetry displays a number of advantages [8]. For example, this image-based technology facilitates high-precision noncontact measurement, and it can handle very large arrays of 3D feature points simultaneously, thus making it suitable for time-constrained deformation measurement of large-scale structures.

Nonexpert user oriented vision metrology systems are well established in industrial metrology [9–13], though application of photogrammetric measuring systems warrants further research and development to make the technology optimally qualified for deformation monitoring of large-scale structures.

Usually, in order to facilitate image measurement, retroreflective targets and highly controlled illumination conditions are employed in vision metrology, and together with the usage of coded target system [14–17], full automation of 3D measurement can be achieved. Recent developments in computer vision put emphasis upon 3D reconstruction of scene structure without the artificial targeting and controlled lighting conditions, which involves extraction of natural feature, feature description, dense matching [18–20], and so forth. These are the developing trends of photogrammetric vision metrology as well, but compared to photogrammetric vision metrology, without targeting, controlled lighting, and coded targets, so far these developments are still far from being fully automated for accurate 3D measurement of large-scale structure.

In this paper, we focus on photogrammetric vision metrology for fully automatic 3D measurement of large-scale structure; all relevant key techniques involved are studied, such as coded target, network orientation, and matching of noncoded target. All will be described in the following sections detailedly.

## 2. Preliminaries

### 2.1. Pinhole Imaging Model

The image point is denoted by a homogeneous 3-vector **x**, and the world point is denoted by a homogeneous 4-vector **X**; then the Pinhole Imaging Model can be simply expressed by

(1) $$\begin{array}{c} ax=K\left[R\;|\;t\right]X, \end{array}$$

where the scalar *a* is an arbitrary scale factor. *K* is a 3 × 3 upper triangular calibration matrix, holding the intrinsic parameters. *R* is a 3 × 3 rotation matrix and **t** is a 3-vector representing the translation vector; *R* and **t** together denote the rigid body transformation between the view and the world coordinate system. The 3 × 4 matrix *P* = *K*[*R* | **t**] is the projection matrix.

### 2.2. Epipolar Geometry

**Figure 1:**
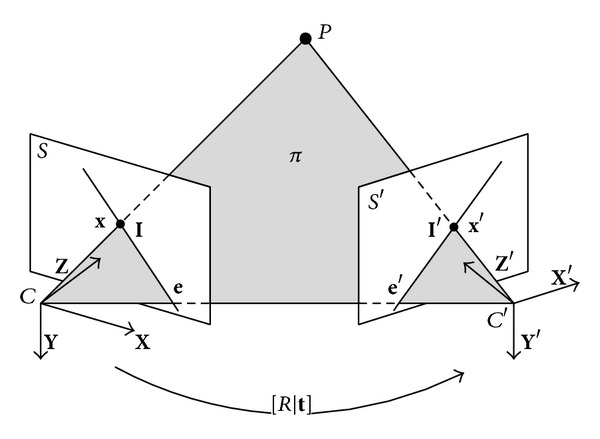
Epipolar geometry.

As shown in Figure 1, a spatial point *P* is projected to **x** and **x**′, respectively, on view *S* and *S*′, obviously, *P*, **x**, **x**′, and two optical centers *C* and *C*′ are in the same spatial plane *π*, this is the well-known coplanarity or epipolar constraint, which is encoded in the 3 × 3 fundamental matrix *F* [21]:

(2) $$\begin{array}{c} x^{\prime T}Fx\;=\;0. \end{array}$$

It can be deduced more intuitively in this way that **x** and **x**′ are located on the epipolar lines **l** = *F* ^*T*^ **x**′ and **l**′ = *F* **x**, respectively, which means **l** ^*T*^ **x** = 0 and **l** ^′*T*^ **x**′ = 0, respectively; both can be rewritten as (2).

The relative pose between view *S* and *S*′ is [*R* | **t**], slightly different from what is stated in 2.1; the coordinate system of view *S*, which is C-XYZ, is now the world coordinate system. Let [**t**]_×_ denote the skew symmetric matrix:

(3) $$\begin{array}{c} {\left[t\right]}_{\times }=\left[\begin{array}{ccc} 0 & -t_{z} & t_{y}\\ t_{z} & 0 & -t_{x}\\ -t_{y} & t_{x} & 0 \end{array}\right], \end{array}$$

then the fundamental matrix *F* is

(4) $$\begin{array}{c} F=K^{\prime -T}{\left[t\right]}_{\times }RK^{-1}, \end{array}$$

where *K* and *K*′ are the calibration matrices of view *S* and *S*′, respectively.

The fundamental matrix *F* can be considered without knowledge of the calibration matrices [21], once *K* and *K*′ are known, which means view *S* and *S*′ are calibrated; image points can be normalized

(5) $$\begin{array}{c} \hat{x}=K^{-1}x,\;\;{\hat{x}}^{\prime }=K^{\prime -1}x^{\prime }, \end{array}$$

where $\hat{x}$ and ${\hat{x}}^{\prime }$ are the normalized image points of **x** and **x**′, respectively; then the epipolar constraint (2) can be simplified to

(6) $$\begin{array}{c} {\hat{x}}^{\prime T}E\hat{x}=0, \end{array}$$

where *E* is the 3 × 3 essential matrix:

(7) $$\begin{array}{c} E={\left[t\right]}_{\times }R. \end{array}$$

### 2.3. Relative Orientation

The mission of relative orientation is to determine the relative pose [*R* | **t**] between two views, as shown in (7); essential matrix *E* contains information about *R* and **t**, so almost every existing relative orientation method, such as the 5-point method [21] and 7- [22] and 8-point methods [22], is based on recovering essential matrix first and then recovering *R* and **t** from it. For simplicity and robustness, the 8-point relative orientation method is adopted in this paper [22].

The projection matrices of *S* and *S*′ are *K*[*I* | 0] and *K*′[*R* | **t**], respectively; once *R* and **t** are recovered, the scene structure can be reconstructed in the coordinate system of *S* by spatial intersection. Preferably, bundle adjustment [23, 24] follows on when relative pose and scene structure are recovered linearly, by which optimal results can be obtained in the least squares sense. Actually, bundle adjustment serves as the last step in almost all motion and structure estimation problems [23].

## 3. Technical Route

Automatic deformation measurement of large-scale structure in this paper is carried out mainly in the following three steps:

- automatic detection of targets, including coded and noncoded ones;
- automatic network orientation using coded targets;
- automatic matching of noncoded targets based on epipolar constraint.

**Figure 2:**
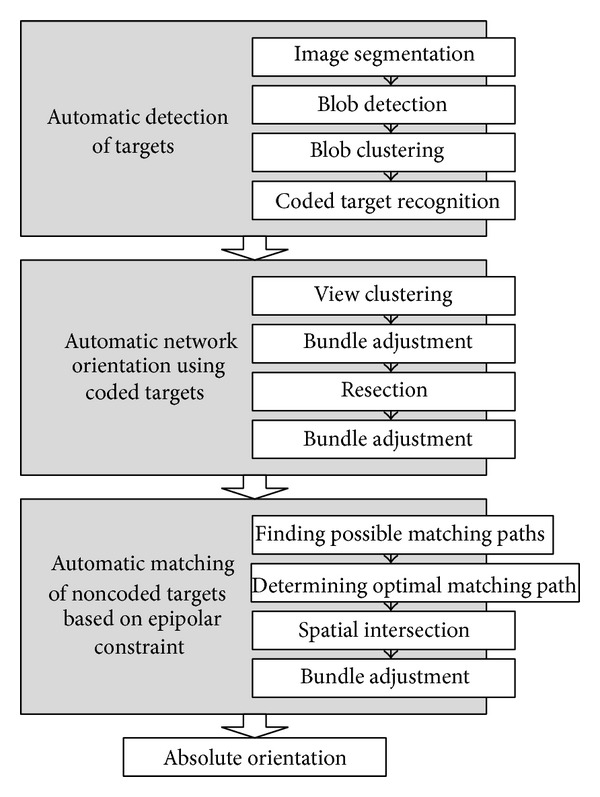
Measuring scheme.

Each step can be further broken down into several detailed substeps; the complete measuring scheme is shown in Figure 2. Key techniques involved are presented in detail in the following sections.

## 4. Design of Coded Target

Coded targets are essential to automation of the whole measuring procedure; they provide point correspondences automatically [25] and ensure reliable results in succeeding network orientation once there is a sufficient number of pairs of homologous points between two images to facilitate relative orientation [25].

Coded target is an automatically detected and recognized pattern of geometric features within the image [14–17]; a qualified coded target is supposed to have sufficient coding capacity and be projective invariant to a certain degree, its corresponding detection algorithm is supposed to be simple, efficient, and robust; moreover, due to measuring accuracy concern, its image points should be able to be measured with high precision.

**Figure 3:**
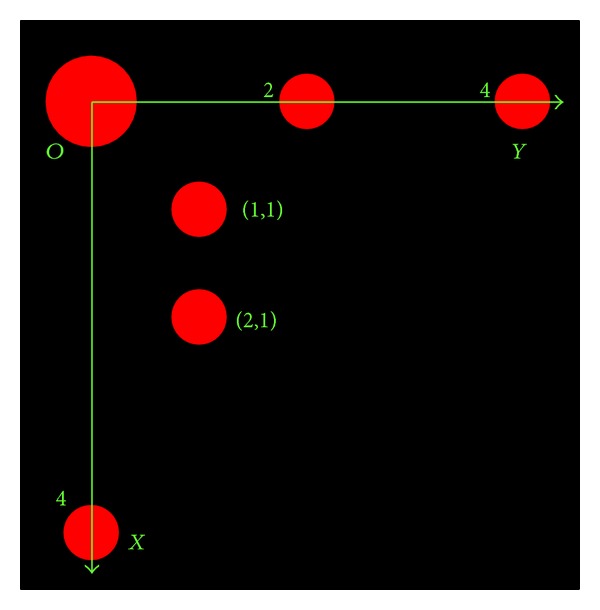
Design of coded target.

The coded target designed in this paper is shown in Figure 3; it consists of several circular retroreflective points, which are arranged according to a certain coding pattern. One reason why circular retroreflective points are adopted is that compared with other geometric features a circular retroreflective point is always imaged as a bright blob under illumination; it is more robust against image degrading processes (e.g., defocusing or changing of the imaging distance and angle) [14]. The other reason is that noncoded target adopted in this paper consists of single circular retroreflective point, like points in coded target; it is also imaged as a bright blob under illumination; thus, identical blob detection algorithm can be applied to coded and noncoded targets at the same time in preliminary stage of detection, avoiding developing different algorithms, respectively.

Out of consideration for detection efficiency, if color images are applied, preferably, the circular retroreflective points in color can be used, just as shown in Figure 3; color information will greatly facilitate the image segmentation, which is essential to detection efficiency.

### 4.1. Coding Principle

There are two kinds of circular points in the designed coded target; one is reference points; there are 4 of them in total, and they together establish the two-dimensional target coordinate system *O*-*XY*, as shown in Figure 3; the biggest among them represents the origin *O* two of the rest represent two different points on *Y*-axis, whose coordinates are (0, 2) and (0, 4), respectively, and the last one represents the point on *X*-axis with coordinates (4, 0).

The other kind is coding points; they are located in positions with positive integer coordinates (*i*, *j*), and each one of them represents one binary bit *k*; the presence and absence of it represent the status 1 and 0 of this binary bit, respectively. If there are *m* coding points at most per column, in other words, the *X* coordinate *i* is not more than *m*, then the relationship between the binary bit *k* and the coordinates (*i*, *j*) is given by

(8) $$\begin{array}{c} k=\left(j-1\right)\times m+i. \end{array}$$

Given that the capacity of coding points per row is *n*, the total amount of binary bits encoded by the designed coded target is *m* × *n*; then the overall coding capacity is 2^*m*×*n*^, which means 2^*m*×*n*^ instances of coded target with different coding information can be derived, for *m* and *n* are chosen arbitrarily; thus, theoretically, there is no upper limit to the coding capacity of the designed coded target.

Take the coded target shown in Figure 3, for example; given that *m* and *n* are both 3, then it represents a binary code with 9 = 3 × 3 binary bits; thus, its coding capacity is 512. There are two coding points in it, with coordinates (1, 1) and (2, 1), respectively; according to (8), they represent the 1st and 2nd binary bits, respectively; the absence of coding points in other 7 positions with positive integer coordinates means the statuses of other 7 binary bits are 0; thus, the encoded binary code is 000000011, and the corresponding decimal code is 2^0^ + 2^1^ = 3.

## 5. Detection of Coded and Noncoded Target

Detection of coded and noncoded targets in this paper is carried out mainly in the following four steps:

- image segmentation simply based on gray information and, preferably, color information, if applicable;
- blob detection based on (Normalized Laplacian of Gaussian) NLoG;
- blob clustering based on image distance between two points; the clusters containing only single blob are determined to be noncoded targets, whereas the clusters containing the required amount of blobs are treated as potential coded targets in succeeding recognition process;
- coded target recognition based on geometric characteristics and coding principle of the designed coded target; potential coded targets cannot be diagnosed until all constraints are met in this recognition process.

### 5.1. Blob Detection

The blob detector adopted in this paper is the well-known scale invariant (Normalized Laplacian of Gaussian) NLoG, which is given as follows:

(9) $$\begin{array}{c} \text{NLoG}\left(x,y;\sigma \right)=\frac{x^{2}+y^{2}-2\sigma^{2}}{2\pi \sigma^{4}}\exp\left(-\frac{x^{2}+y^{2}}{2\sigma^{2}}\right), \end{array}$$

where *σ* is the scale parameter; and the detection of blobs with multiple scales is to detect scale-space maxima/minima, which are points that are simultaneously local maxima/minima with respect to both space and scale [26, 27].

### 5.2. Blob Clustering

As stated before, a coded target is imaged as a group of blobs, whereas a noncoded one is imaged as one single blob, which means the blobs within a coded target distributes much denser than blobs of multiple noncoded targets do; this will not change as long as the distribution density of multiple noncoded targets in space does not exceed the one of points within a coded target, and the depths of noncoded targets, relative to camera, do not vary too much from the one of a coded target. Thus, simple blob clustering based on image distance between two points can be conducted to distinguish between coded and noncoded targets.

**Figure 4:**
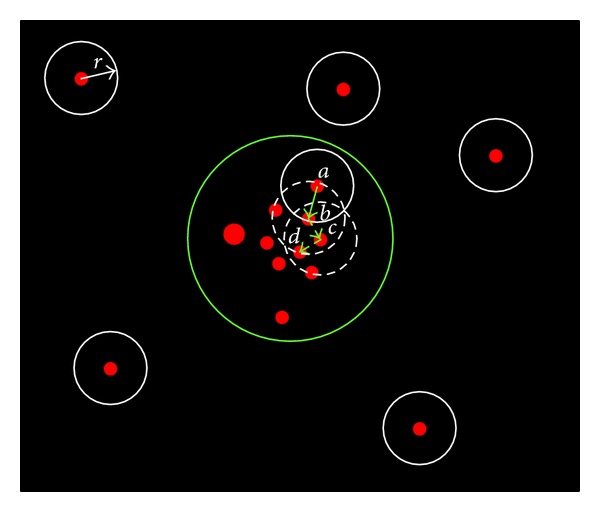
Blob clustering process.

The blob clustering process is demonstrated in Figure 4. The group of blobs surrounded by the big green circle is a coded target, and the rest blobs are all noncoded ones. The white circle surrounding each blob with radius *r* denotes the adjacent area of each blob; if a blob is within this adjacent area of another blob, then these two blobs are adjacent to each other and can be clustered into the same cluster. As you can see, there are no other blobs within the adjacent area of each noncoded target, so the cluster of each noncoded target contains only its own blob.

Let us check out step by step how clustering works out for coded target; if it starts at blob *a* first, blob *b* will be added into cluster next, for it is within the adjacent area of blob *a*, and then come blobs *c* and *d*; clustering will keep going until no more adjacent blobs are found.

**Figure 5:**
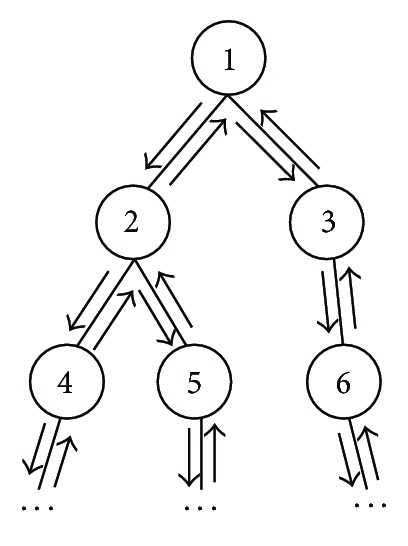
Recursive routine.

This blob clustering process, like the view clustering process which is going to be described in the succeeding section, is a standard recursive process; they both can be implemented by the recursive routine shown in Figure 5.

After clustering, clusters containing only single blob are determined to be noncoded targets directly, and clusters containing reasonable amount of blobs are determined to be potential coded targets, since a true coded target contains at least 4 reference points and limited amount of coding points. If a cluster contains unreasonable amount of blobs, blobs within it will be sentenced to be noncoded targets as well.

### 5.3. Coded Target Recognition

As stated before, coded target recognition is based on geometric characteristics and coding principle of the designed coded target; potential coded targets coming from blob clustering cannot be diagnosed until all constraints are met in this recognition process. Coded target recognition is carried out mainly in the following two steps:

- recognition of reference points;
- decoding.

#### 5.3.1. Recognition of Reference Points

**Figure 6:**
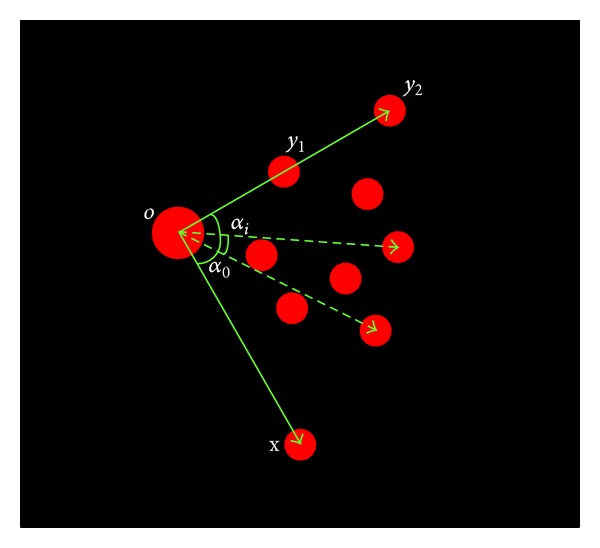
Recognition of reference points.

The first thing to do is to determine which blob is the origin *O* of the target coordinate system. As shown in Figure 6, the origin *O* is much bigger than any of the other blobs, so it can be located simply by finding the blob with the biggest size. Naturally, radius can be considered; it is a measure of blob size, and it is readily acquired in blob detection stage, but radius alone cannot ensure clear distinction between the origin *O* and other blobs, since the radius difference between two blobs may be smaller than the scale resolution in blob detection, which is the scale step between two NLoG templates; thus, more robust method is needed. In this paper, the grayscale sum of pixels within blob radius, in other words, the grayscale weighted area, is used to make a clear distinction of the origin *O* from other blobs; the blob with the biggest grayscale weighted area is determined to be the origin *O*.

The rest three reference points, *x*, *y*_1_, and *y*_2_, have their own geometric characteristics; the angle between segments *Oy*_1_ and *Ox* equals the angle between *Oy*_2_ and *Ox*, which is both *α*_0_ as shown in Figure 6; besides, these two angles are bigger than angle *α*_*i*_ formed by any other two blobs toward origin *O*; based on these, *x*, *y*_1_, and *y*_2_ are determined in this paper by finding angle *α*_0_.

#### 5.3.2. Decoding

Technically, the relationship between the image coordinates of a coding point, which is (*x*, *y*), and its target coordinates (*i*, *j*) is a projective transformation; however, 4 pairs of correspondences between image and target generated by 4 reference points are unable to solve this projective transformation, since there are 3 reference points which are collinear, which are *O*, *y*_1_, and *y*_2_, respectively. Fortunately, when target depth, relative to camera, is far greater than target size, as is usually the case in practice, the relationship between (*x*, *y*) and (*i*, *j*) approximates to an affine transformation *H*_*a*_:

(10) $$\begin{array}{c} \left(\begin{array}{c} j\\ i\\ 1 \end{array}\right)=\left(\begin{array}{ccc} a_{1} & a_{2} & a_{3}\\ a_{4} & a_{5} & a_{6}\\ 0 & 0 & 1 \end{array}\right)\left(\begin{array}{c} x\\ y\\ 1 \end{array}\right). \end{array}$$

At least 3 pairs of correspondences, generated by 3 noncollinear points, are adequate to solve *H*_*a*_; thus, the recognized 4 reference points in Section 5.3.1 can be used to solve *H*_*a*_.

Once *H*_*a*_ is recovered, the target coordinates (*i*, *j*) of each coding point can be computed according to (9), and corresponding binary bit *k* of each coding point can be further acquired according to (8); then the binary and decimal code is finally decoded.

## 6. Automatic Network Orientation

The mission of automatic network orientation is to automatically determine the relative pose between each view and certain reference frame, which is usually the coordinate system of the first view, and reconstruct all coded targets at the same time. A three-stage automatic network orientation strategy is proposed in this paper:

- view clustering based on relative orientation with the help of coded targets and reconstruction of coded targets at the same time;
- connecting those view clusters which contain multiple views using absolute orientation;
- conducting resections for isolate views, which cannot be clustered with other views, using coded targets reconstructed in view clustering.

### 6.1. View Clustering

As stated in [28], many researches on structure and motion recovery have been based on some certain image ordering, usually in chronological order if image set is sequential; this image ordering allows small baseline matching algorithms to be used between consecutive frames of the sequence and avoids wide baseline situations, in which matching is difficult; thus, all images can be successfully sewed together into tracks image by image.

In our case, the usage of coded targets greatly alleviates our concerns with matching; it can provide reliable point correspondences between views, even in wide baseline situations for coded target itself is robust to large perspective distortions, as stated in Sections 4 and 5. Yet this ordering remains advantageous to our case, since the small baseline conditions it brings mean bigger overlapping area between footprints of consecutive views, in other words, more point correspondences available for relative orientation. But sometimes this ordering is not guaranteed in our case, in which way relative orientation between views may fail for overlapping area between footprints of these views is not big enough to provide adequate correspondences; in order to cope with this situation and automate the network orientation process, view clustering is carried out.

Unlike view clustering in [28], in which feature matching is involved, view clustering in our case is much simpler due to the usage of coded targets; whether two views can be clustered into one group or not simply depends on whether the relative orientation between these two views succeeds or fails.

As stated in Section 5.2, like the blob clustering, view clustering process is also implemented by the recursive routine shown in Figure 5.

**Figure 7:**
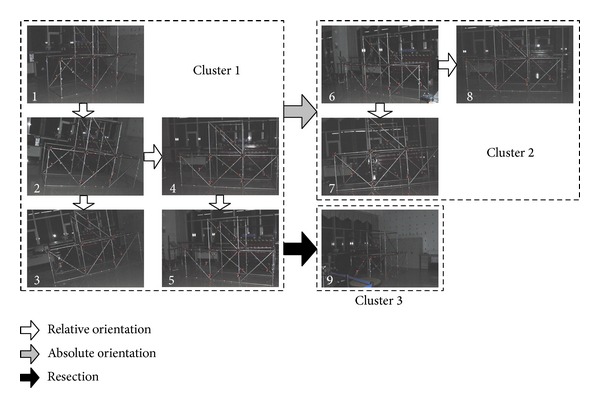
Network orientation process.

The whole network orientation process is demonstrated in Figure 7 by images of a steel structure, as shown; there are 9 views in total, and three clusters emerge after view clustering. The first five are clustered together to be cluster 1, view 6 to view 8 form the cluster 2, and view 9 is left alone to be cluster 3.

If at least three homologous coded targets are reconstructed both in cluster 1 and cluster 2, then cluster 2 can be connected to cluster 1 using absolute orientation.

As for view 9, it is an isolate view for it observes only five coded targets, which is insufficient to carry out relative orientation between it and any other views, but connection can still be built between it and cluster 1 by resection, as long as there are adequate observed coded targets, which have already been reconstructed in cluster 1, the minimum amount required for resection can be at least 4 under certain circumstances.

Be aware that bundle adjustment is recommended as the last step in all three stages of network orientation.

## 7. Matching of Noncoded Target

Unlike coded target, noncoded target cannot provide its matches over multiple views by itself; besides, feature descriptor based methods [18–20] are not suitable for matching of noncoded targets in our case, where the illumination is highly controlled to ensure that all targets can be readily detected; for the same reason, images in our case are usually textureless, which makes feature descriptor not applicable.

Given that all views that can be oriented have already been automatically oriented in network orientation, noncoded targets can be matched over views based on epipolar constraints, introduced in Section 2.2, and this is also how it is done in this paper but in a subtler way. The concept of matching paths is proposed, and matching of noncoded targets in this paper is carried out mainly in two steps:

- finding all possible matching paths through multiple views for each noncoded target;
- determining the optimal matching path for each noncoded target by a novel voting strategy.

### 7.1. Finding Possible Matching Paths

**Figure 8:**
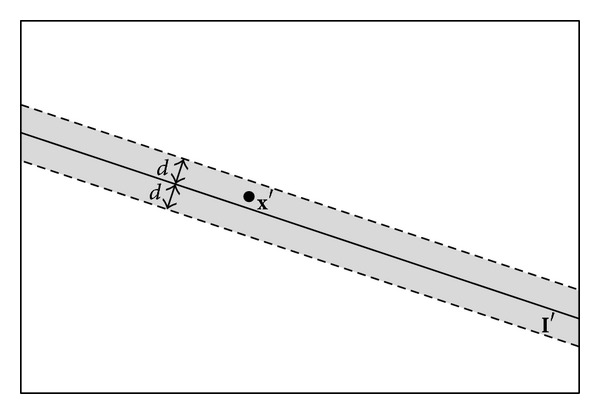
Determining potential match based on epipolar constraint.

As stated in Section 2.2 and shown in Figure 1, given the fundamental matrix *F*, the match in view *S*′, which is **x**′, for a given **x** in view *S*, is theoretically located on the epipolar line **l**′ = *F* **x**, which means **l** ^′*T*^ **x**′ = 0, and this is what our matching strategy is based on. But when the estimate for *F* and the measurements of **x** and **x**′ are not error free, as is the case in practice, **x**′ will not be exactly on **l**′ anymore but close to it; in other words, **l** ^′*T*^ **x**′ ≠ 0; thus, in practice once the image distance between **x**′ and **l**′ is smaller than certain threshold *d*, as shown in Figure 8, **x**′ will be determined to be a potential match for **x**.

**Figure 9:**
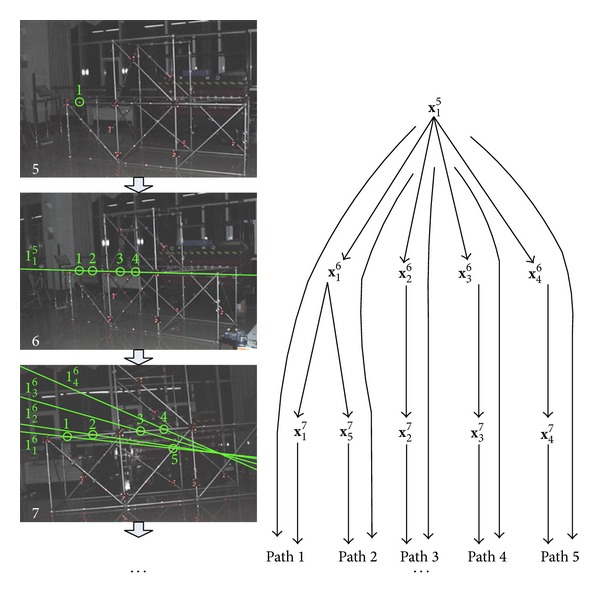
Finding possible matching paths.

Sometimes, besides *P* there will be other spatial points in the plane *π* as well, as shown in Figure 1; this will bring ambiguity to finding match for **x**, since projections of these points on view *S*′ are also on **l**′. In such cases, when we try to determine which image point on *S*′ is the match for **x** based on criterion shown in Figure 8, we will be expected to find multiple candidates rather than one, just as shown in Figure 9; four potential matches in view 6 for point 1 in view 5 are found.

Obviously, if there is a third view with optical center out of the plane *π*, this ambiguity will be eliminated. But in order to reduce complexity and increase the automaticity and robustness, we do not explicitly attempt to find this third view during matching in this paper; instead, a simpler and subtler way is adopted. We first maintain the ambiguity and find all possible matching paths through multiple views for a given point, then determine the optimal path based on a novel voting strategy, eliminating the ambiguity, and finally find the real match.

A matching path is defined as follows:

(11) $$\begin{array}{c} \text{path}\left(x_{j_{1}}^{i_{1}},x_{j_{2}}^{i_{2}},\ldots ,x_{j_{k}}^{i_{k}},\ldots ,x_{j_{w}}^{i_{w}}\right),\\ 1\leq k\leq w,\;1\leq i_{k}\leq m,\;1\leq j_{k}\leq n\left(i_{k}\right), \end{array}$$

where **x**_*j*_*k*__^*i*_*k*_^ denotes the *k*th matching image point in the matching path, *i*_*k*_ is the view number of this point, or in which view it is measured, *j*_*k*_ is its point number among all observations in view *i*_*k*_, *w* is the total amount of points contained in the path, *m* denotes the total amount of views, and *n*(*i*_*k*_) represents the total amount of observations in view *i*_*k*_.

The process of finding possible matching paths for a given image point is demonstrated in Figure 9 still by images of a steel structure. As shown, the point 1 in view 5, which is **x**_1_^5^, finds four matches in view 6, resulting in four separate paths; then we continue to find matches in view 7 for those four points. Since the point 1 in view 6, which is **x**_1_^6^, can find two different matches in view 7, a previous path now splits into two, resulting in five separate paths in total, which are

(12) $$\begin{array}{c} \text{path }1\left(x_{1}^{5},x_{1}^{6},x_{1}^{7}\right)\\ \text{path }2\left(x_{1}^{5},x_{1}^{6},x_{5}^{7}\right)\\ \text{path }3\left(x_{1}^{5},x_{2}^{6},x_{2}^{7}\right)\\ \text{path }4\left(x_{1}^{5},x_{3}^{6},x_{3}^{7}\right)\\ \text{path }5\left(x_{1}^{5},x_{4}^{6},x_{4}^{7}\right). \end{array}$$

Given that *F*_*b*_^*a*^ is the fundamental matrix between view *a* and view *b*, then the epipolar line **l**_*j*_^*a*^ in view *b*, as shown in Figure 9, is

(13) $$\begin{array}{c} l_{j}^{a}=F_{b}^{a}x_{j}^{a}, \end{array}$$

where **x**_*j*_^*a*^ is the point *j* in view *a*. In short, **l**_*j*_^*a*^ is an epipolar line induced by **x**_*j*_^*a*^.

As the process described above continues, the amount of matching paths will be expected to increase, but if the amount of paths remains one eventually, image points contained in the only matching path will naturally be determined to be matched or the voting strategy, which is described in the next section, will step in.

### 7.2. Determining Optimal Matching Path

The novel voting strategy proposed in this paper is still based on epipolar constraint; if image points contained in a matching path are really matched, epipolar constraint should be met between all N-choose-2 points; thus, we can determine whether a path is the real one simply by checking if all the N-choose-2 cases in it meet the epipolar constraint. However, due to error, probably not all the cases are qualified, so in practice it is reasonable to choose the path with most qualified cases instead of all qualified cases as the optimal matching path, which contains points really matched.

The vote that a matching path can get is given by

(14) $$\begin{array}{c} \text{vote}=\sum_{k_{1}=1\;}^{w}\sum_{k_{2}=1}^{w}\text{check}\left(x_{j_{k_{1}}}^{i_{k_{1}}},x_{j_{k_{2}}}^{i_{k_{2}}}\right),\;k_{2}\neq k_{1}, \end{array}$$

where **x**_*j*_*k*_1___^*i*_*k*_1__^ and **x**_*j*_*k*_2___^*i*_*k*_2__^ are, respectively, the *k*_1_th and *k*_2_th points in the path, and the function check is given as follows:

(15) $$\begin{array}{c} \text{check}\left(x_{j_{k_{1}}}^{i_{k_{1}}},x_{j_{k_{2}}}^{i_{k_{2}}}\right)=\{\begin{array}{cc} 1 & \text{if}\;\mathrm{dist}\left(x_{j_{k_{1}}}^{i_{k_{1}}},F_{i_{k_{1}}}^{i_{k_{2}}}x_{j_{k_{2}}}^{i_{k_{2}}}\right)<d\\ 0 & \text{else}, \end{array} \end{array}$$

where dist⁡(**x**_*j*_*k*_1___^*i*_*k*_1__^, *F*_*i*_*k*_1___^*i*_*k*_2__^ **x**_*j*_*k*_2___^*i*_*k*_2__^) is the distance between point **x**_*j*_*k*_1___^*i*_*k*_1__^ and the epipolar line *F*_*i*_*k*_1___^*i*_*k*_2__^ **x**_*j*_*k*_2___^*i*_*k*_2__^ and *d* is some certain distance threshold, as explained in Section 7.1. In short, the function check means that if the *k*_1_th point is close enough to the epipolar line in view *i*_*k*_1__ induced by the *k*_2_th point, the path gets one vote.

Apparently, each N-choose-2 case has two votes to give, which is check(**x**_*j*_*k*_1___^*i*_*k*_1__^, **x**_*j*_*k*_2___^*i*_*k*_2__^) and check(**x**_*j*_*k*_2___^*i*_*k*_2__^, **x**_*j*_*k*_1___^*i*_*k*_1__^), respectively, so if all N-choose-2 cases in a path meet the epipolar constraint, this path will get a unanimous vote, which is *w*(*w* − 1).

Taking the three images shown in Figure 9 as an example again and using the voting strategy described above, path 1 gets the most votes, which is 6, whereas all other paths get only 4 votes, so path 1 is chosen to be the optimal matching path up to this point, just as what it really is; thus, the points contained in path 1 are determined to be matched.

## 8. Experimental Results

**Figure 10:**
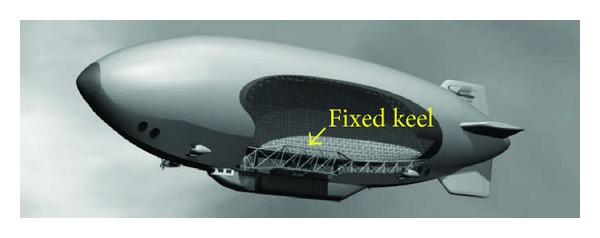
Semirigid airship.

The main types of airship are nonrigid blimps, semirigid airship, and rigid airship. Unlike the nonrigid blimps, semirigid airship usually has a fixed keel besides internal pressure, which runs the length of the ship along the bottom of the hull, as shown in Figure 10, and provides greater structural strength to maintain its shape and structural integrity.

**Figure 11:**
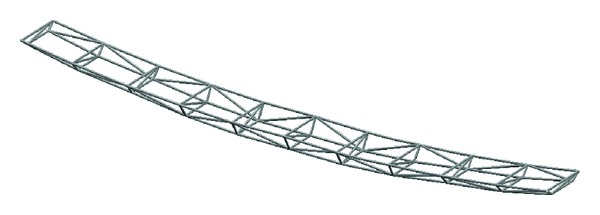
Designed keel structure.

**Figure 12:**
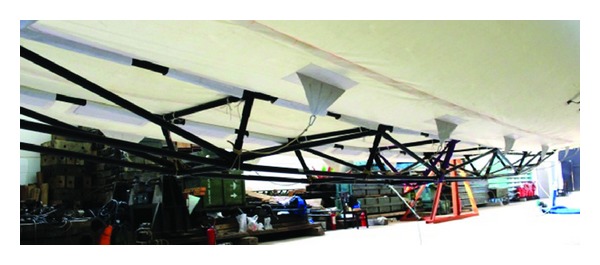
Designed keel structure in field.

A designed keel structure in a practical semi-airship is shown in Figures 11 and 12.

In order to ensure safety and prevent potential structural failure in extreme conditions like storm, it is necessary to conduct mechanical testing and high-precision dimensional quality monitoring for this designed keel structure before the airship is put into use.

**Figure 13:**
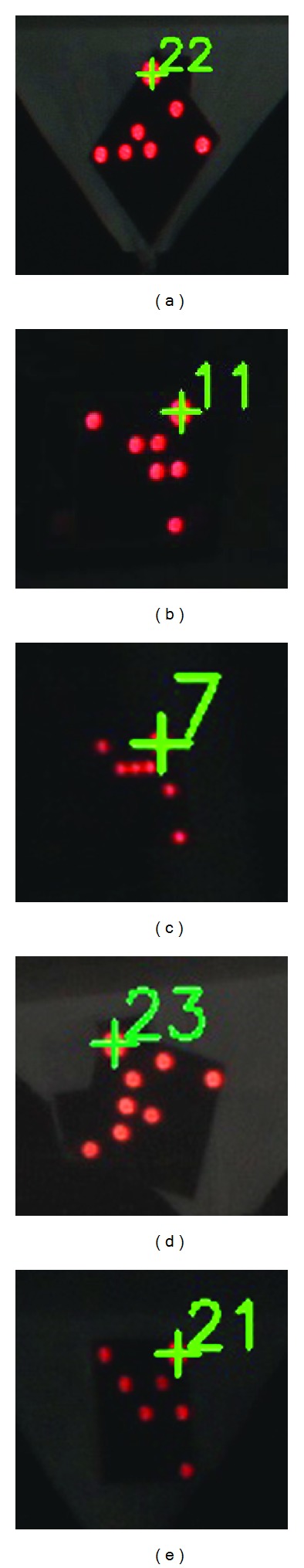
Detection of coded targets.

**Figure 14:**
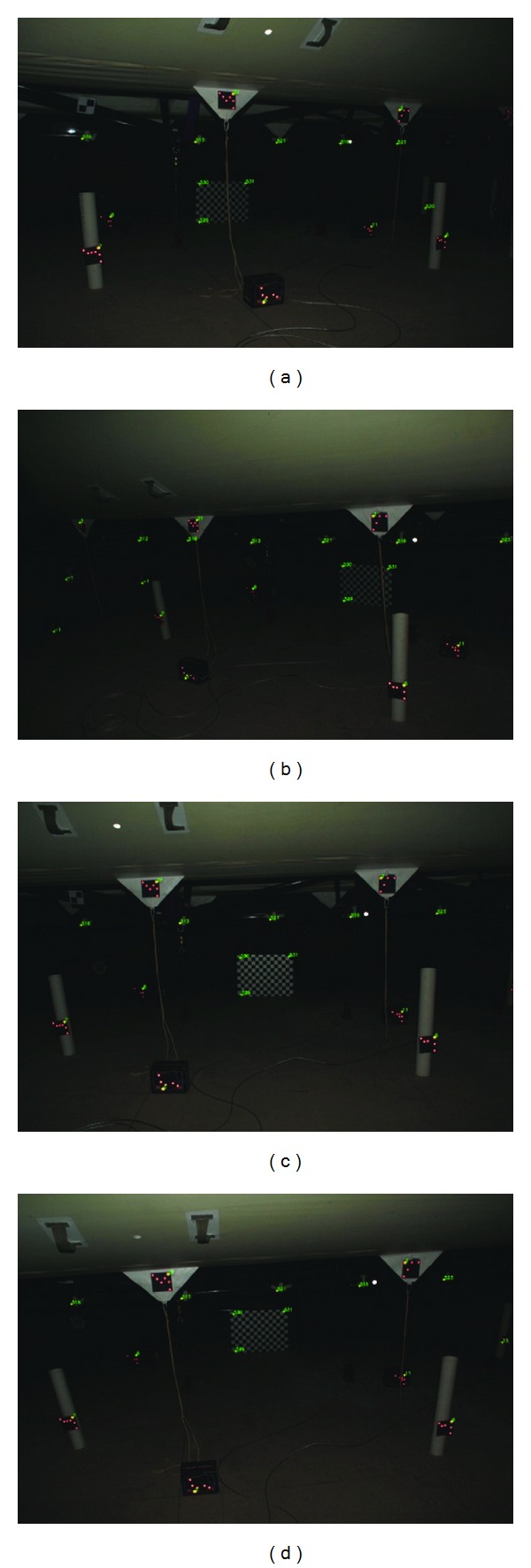
Images of scene structure.

The proposed methods in this paper have been applied to this mechanical testing, and results are shown in the following figures. The reference frame is established by the three points on the chessboard shown in Figure 14, with origin located on the top left corner of the chessboard. Red noncoded targets are attached to the main axis of the keel node by node, with the positional changes of these noncoded targets reflecting the deformations of the keel under different loads and internal pressures. Red coded targets are scattered evenly in the scene to facilitate network orientation; examples of detection of coded targets are given in Figure 13, and as you can see, detection of coded targets is quite robust to large perspective distortions.

**Figure 15:**
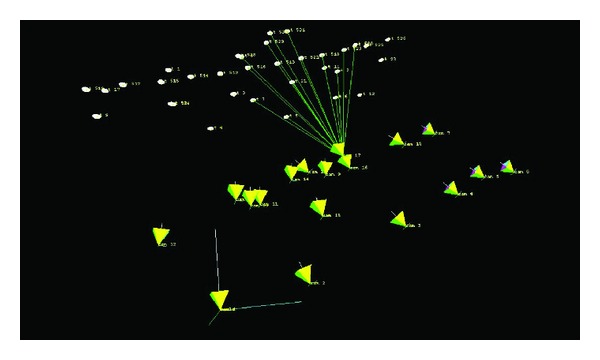
Recovery of camera motion and scene structure.

There are 18 images which are taken under one working condition; some of these images are shown in Figure 14, and the recovered camera motion and scene structure are shown in Figure 15.

In order to verify the measuring accuracy, vertical deformations of one node under different load and internal pressure conditions are measured by total station besides our automatic vision metrology as true values, and the deviations of our measurement results from the true values are given in Table 1. As you can see, measuring errors under all working conditions are within 1 mm, which is considerably good.

## 9. Summary and Conclusions

In this paper, all relevant key techniques involved in photogrammetric vision metrology for fully automatic 3D measurement of large-scale structure are studied. A new kind of coded target consisting of circular retroreflective discs is designed, and corresponding detection and recognition algorithms based on blob detection and clustering are presented. Then a three-stage strategy starting with view clustering is proposed to achieve automatic network orientation. As for matching of noncoded targets, the concept of matching path is proposed, and matches for each noncoded target are found by determination of the optimal matching path, based on a novel voting strategy, among all possible ones. Experiments on a fixed keel of airship have been conducted to verify the effectiveness and measuring accuracy of the proposed methods.

Future work will mainly focus on developing new blob clustering method that is more adaptive to coded targets in different depths, and improved method that is less sensitive to starting point should be developed for finding all possible matching paths, since, in current way, different starting points may lead to different amount of points within the optimal paths with some matched points missed.

## Figures and Tables

**Table 1:** Measurement deviations.

Working conditions	1 kN/500 Pa	1 kN/700 Pa	2 kN/500 Pa	2 kN/700 Pa	3 kN/500 Pa	3 kN/700 Pa
Total station	21.78 mm	21.62 mm	46.05 mm	42.69 mm	66.42 mm	61.83 mm
Ours	22.07 mm	21.90 mm	46.61 mm	43.27 mm	67.22 mm	62.52 mm
Error	0.29 mm	0.28 mm	0.56 mm	0.58 mm	0.80 mm	0.69 mm
